# Supplementary material for: HBO1-MLL interaction promotes AF4/ENL/P-TEFb-mediated leukemogenesis
Source: eLife. 2021 Aug 25;10:e65872. doi: 10.7554/eLife.65872 (PMC8387021; doi:10.7554/eLife.65872)

Figure 2

B

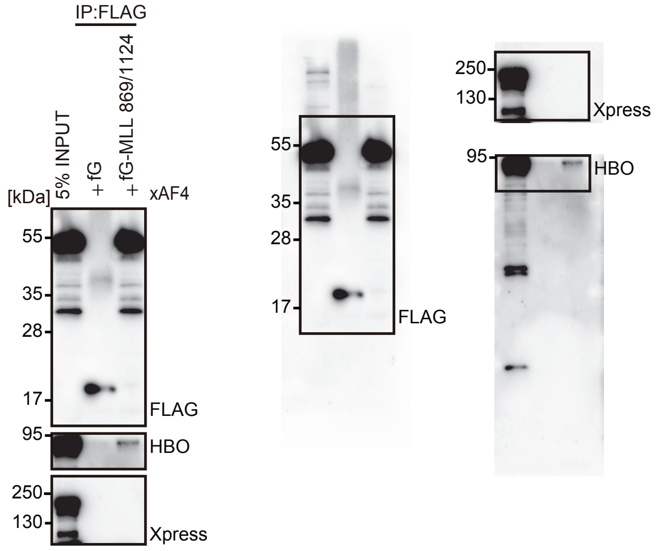

C

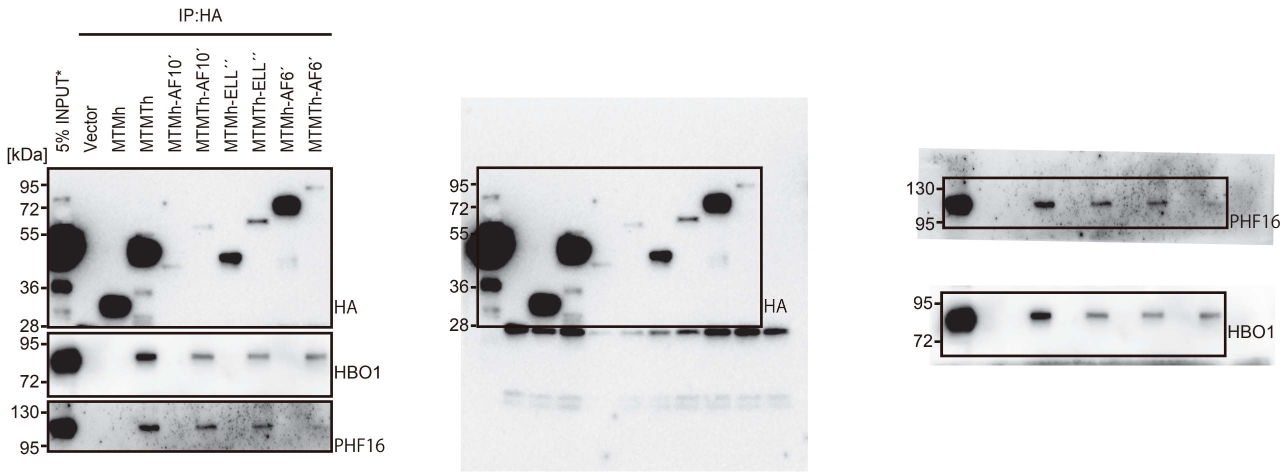

Figure 3

D

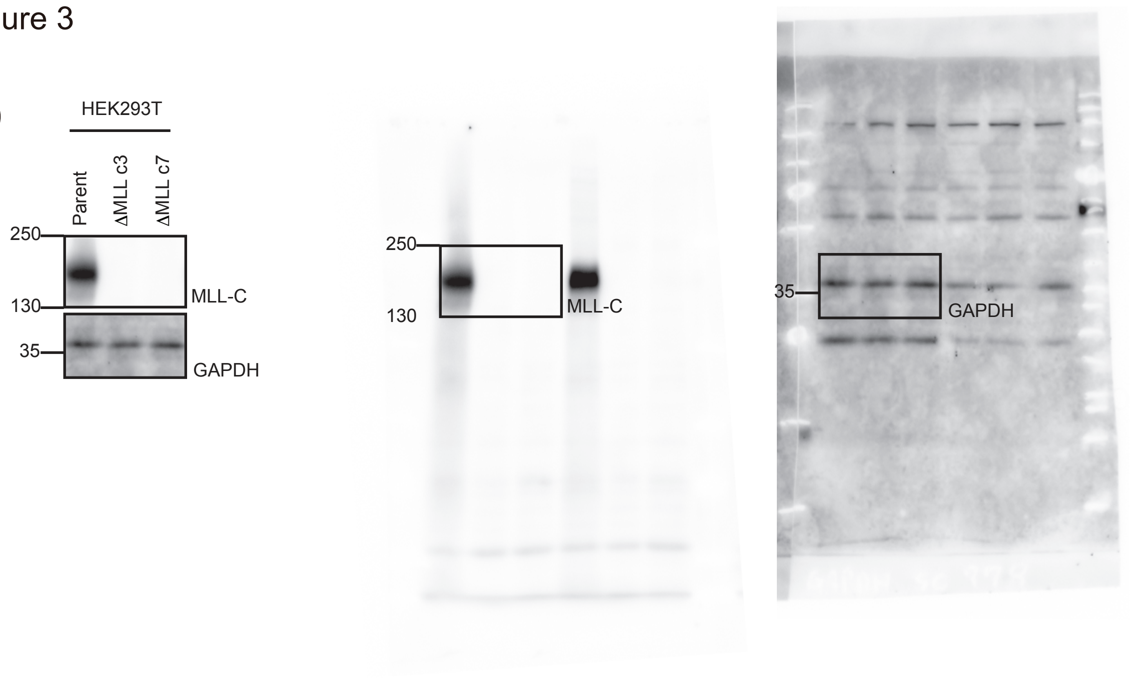

Figure 4

B

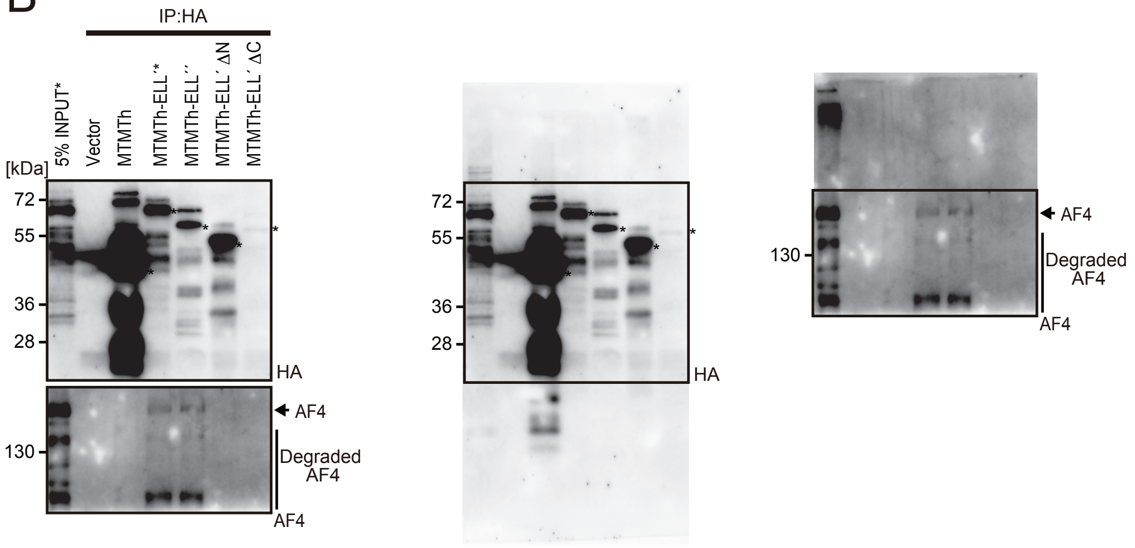
IP:HA
5% INPUT*
Vector
MTMTh
MTMTh-ELL´*
MTMTh-ELL´´
MTMTh-ELL´ ΔN
MTMTh-ELL´ ΔC
[kDa]
72
55
36
28
HA
AF4
130
Degraded AF4
AF4

D

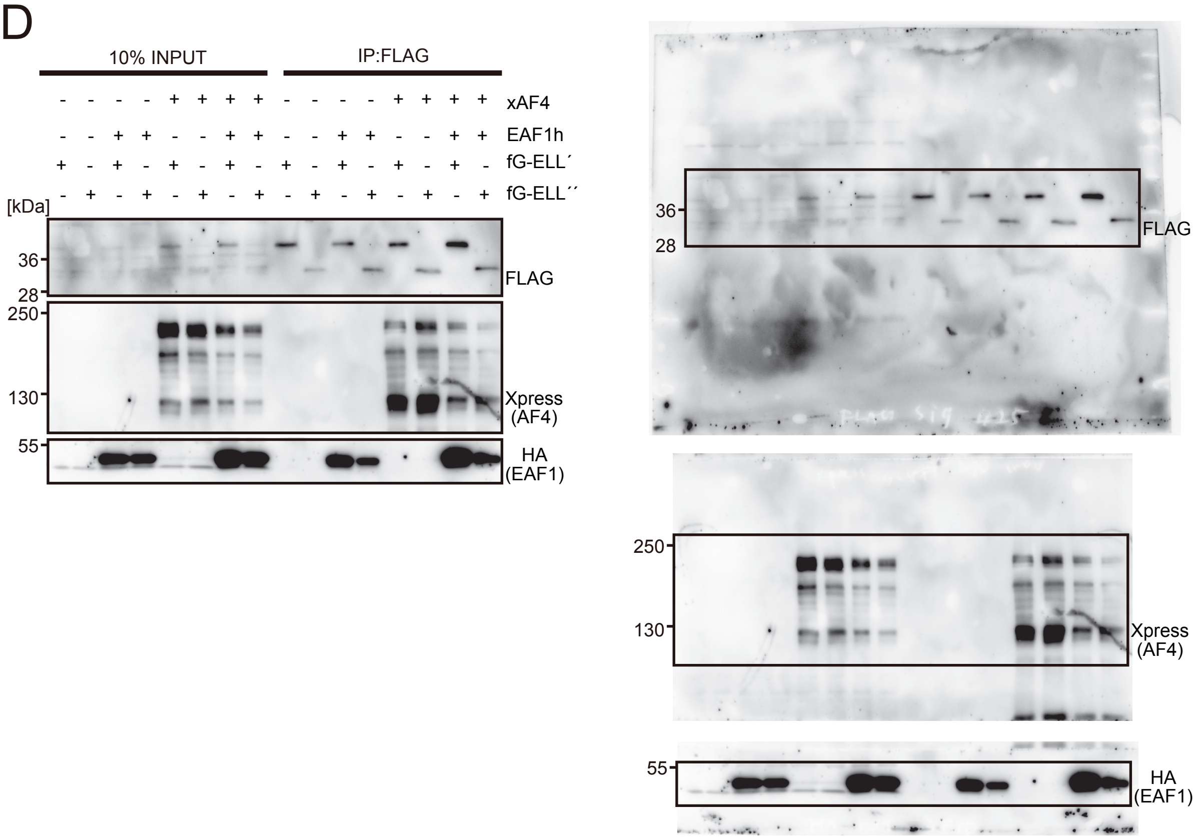
10% INPUT
IP:FLAG
xAF4
EAF1h
fG-ELL´
fG-ELL´´
[kDa]
36
28
FLAG
250
130
Xpress (AF4)
55
HA (EAF1)

Figure 5

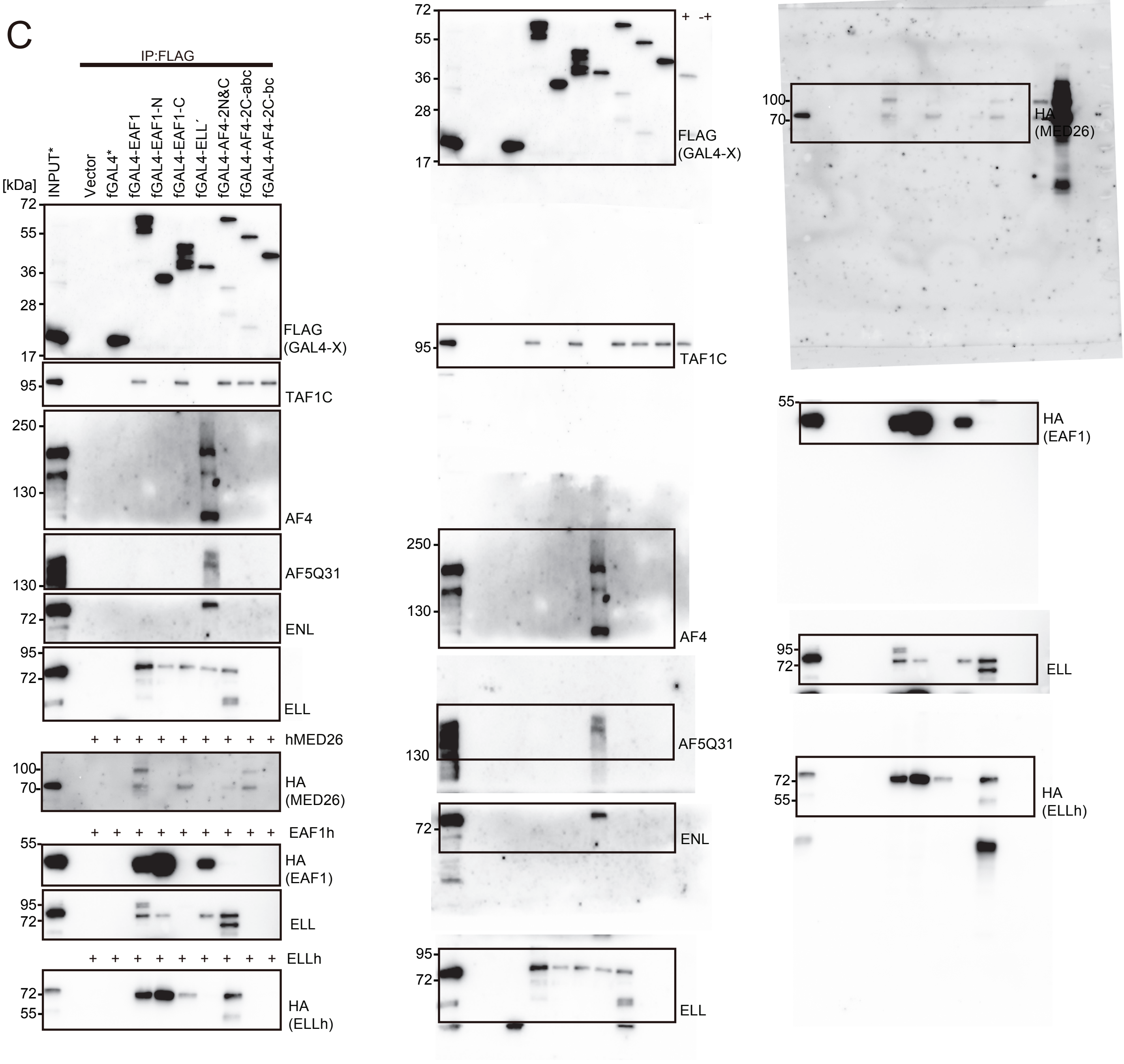
C
IP:FLAG
[kDa]
INPUT*
Vector
fGAL4*
fGAL4-EAF1
fGAL4-EAF1-N
fGAL4-EAF1-C
fGAL4-ELL´
fGAL4-AF4-2N&C
fGAL4-AF4-2C-abc
fGAL4-AF4-2C-bc
FLAG (GAL4-X)
TAF1C
AF4
AF5Q31
ENL
ELL
hMED26
HA (MED26)
EAF1h
HA (EAF1)
ELLh
HA (ELLh)
72
55
36
28
17
95
250
130
100
70

Figure 6

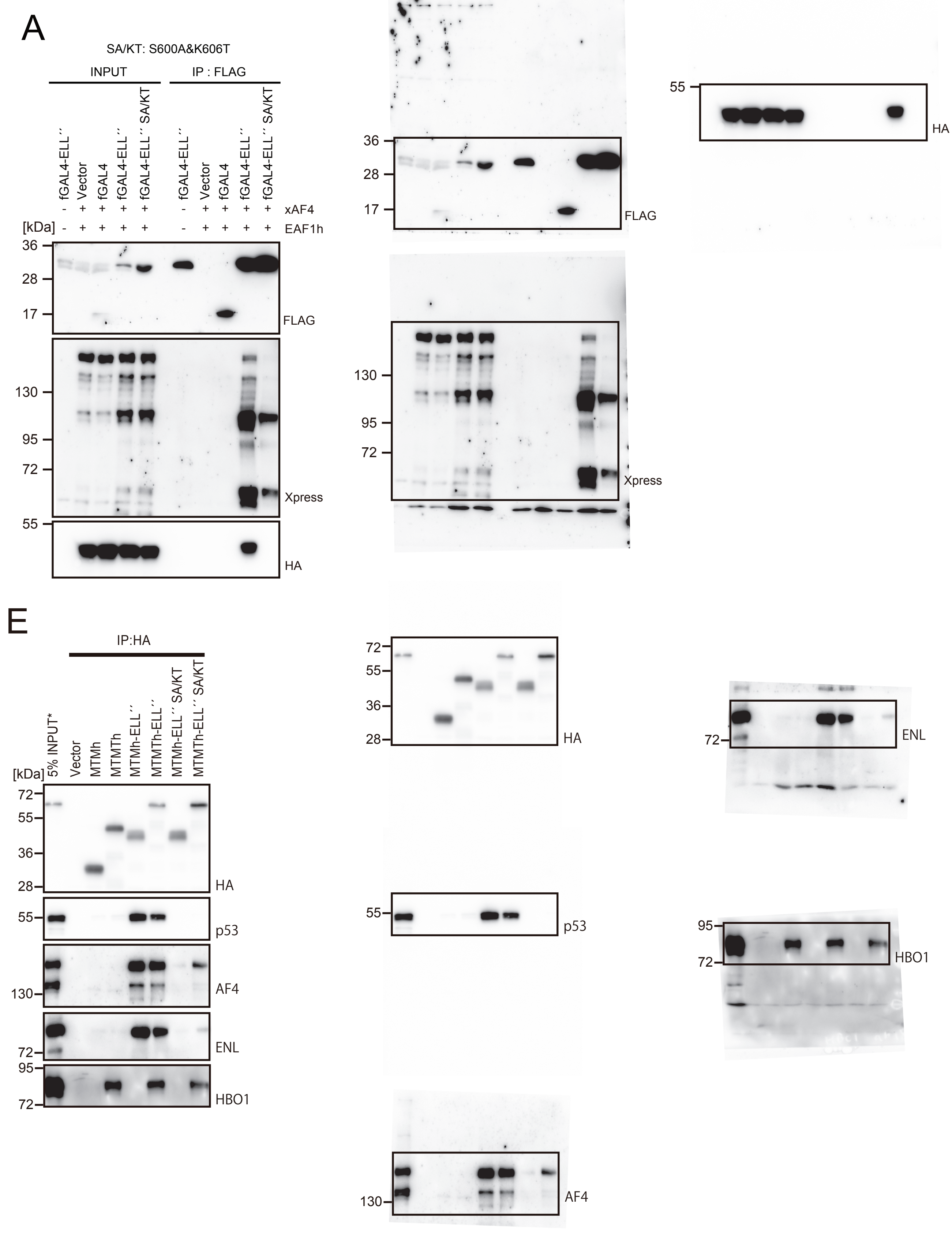
A
SA/KT: S600A&K606T
INPUT
IP : FLAG
fGAL4-ELL´´
Vector
fGAL4
fGAL4-ELL´´
fGAL4-ELL´´ SA/KT
xAF4
EAF1h
[kDa]
FLAG
Xpress
HA
E
IP:HA
5% INPUT*
Vector
MTMh
MTMTh
MTMh-ELL´´
MTMTh-ELL´´
MTMh-ELL´´ SA/KT
MTMTh-ELL´´ SA/KT
HA
p53
AF4
ENL
HBO1

Figure 7

B

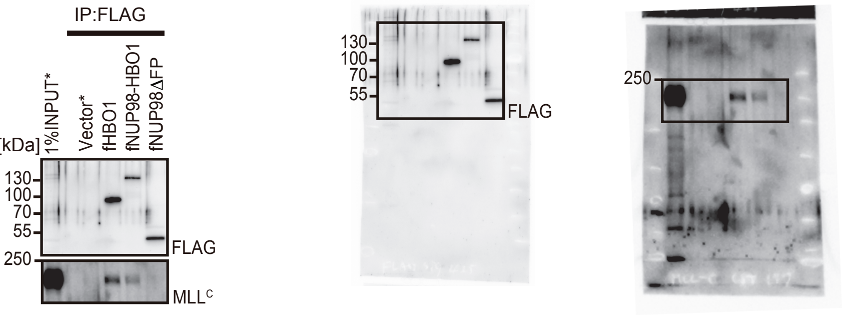
IP:FLAG
1%INPUT*
Vector*
fHBO1
fNUP98-HBO1
fNUP98ΔFP
[kDa]
130
100
70
55
250
FLAG
MLLC
130
100
70
55
FLAG
250

D

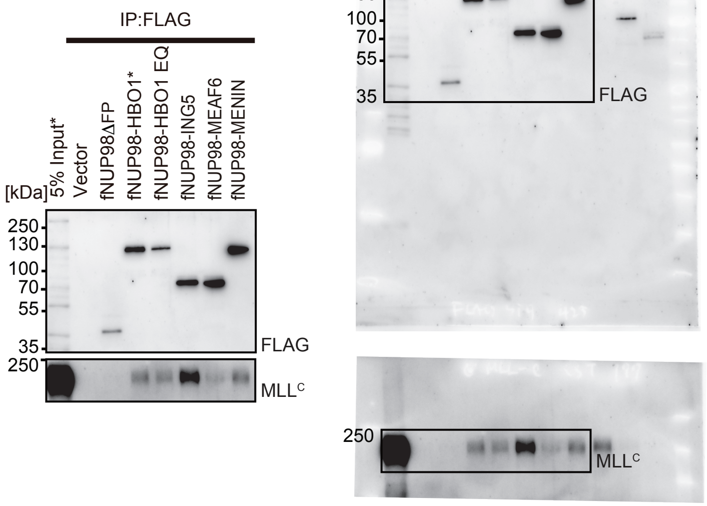
IP:FLAG
5% Input*
Vector
fNUP98ΔFP
fNUP98-HBO1*
fNUP98-HBO1 EQ
fNUP98-ING5
fNUP98-MEAF6
fNUP98-MENIN
[kDa]
250
130
100
70
55
35
FLAG
250
MLLC
250
130
100
70
55
35
FLAG
250
MLLC

Figure 8

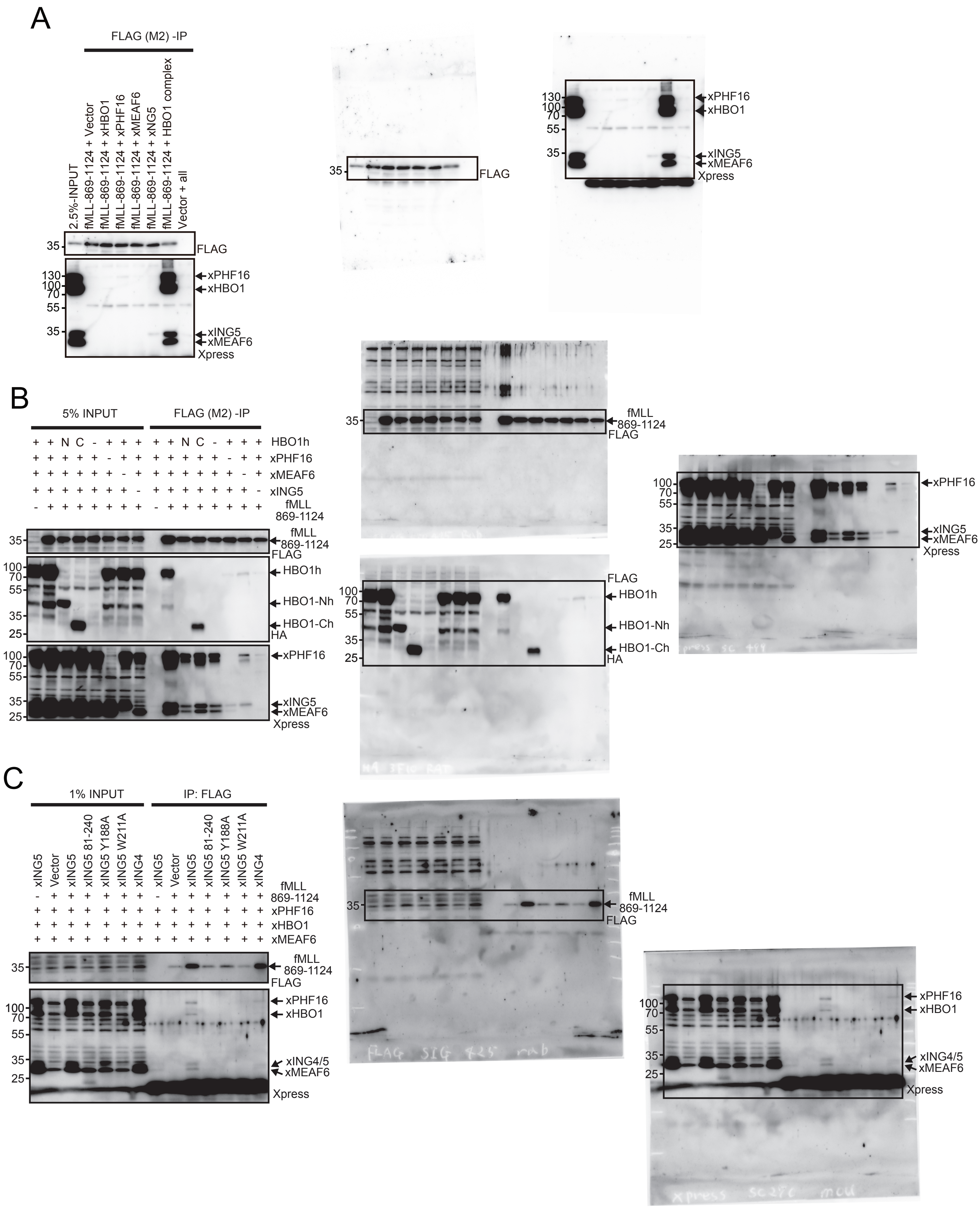
A
FLAG (M2) -IP
2.5%-INPUT
fMLL-869-1124 + Vector
fMLL-869-1124 + xHBO1
fMLL-869-1124 + xPHF16
fMLL-869-1124 + xMEAF6
fMLL-869-1124 + xNG5
fMLL-869-1124 + HBO1 complex
Vector + all
FLAG
xPHF16
xHBO1
xING5
xMEAF6
Xpress
B
5% INPUT
FLAG (M2) -IP
HBO1h
xPHF16
xMEAF6
xING5
fMLL 869-1124
FLAG
HBO1h
HBO1-Nh
HBO1-Ch
HA
xPHF16
xING5
xMEAF6
Xpress
C
1% INPUT
IP: FLAG
xING5
Vector
xING5
xING5 81-240
xING5 Y188A
xING5 W211A
xING4
fMLL 869-1124
xPHF16
xHBO1
xMEAF6
FLAG
xPHF16
xHBO1
xING4/5
xMEAF6
Xpress

Figure 1-figure supplement 1

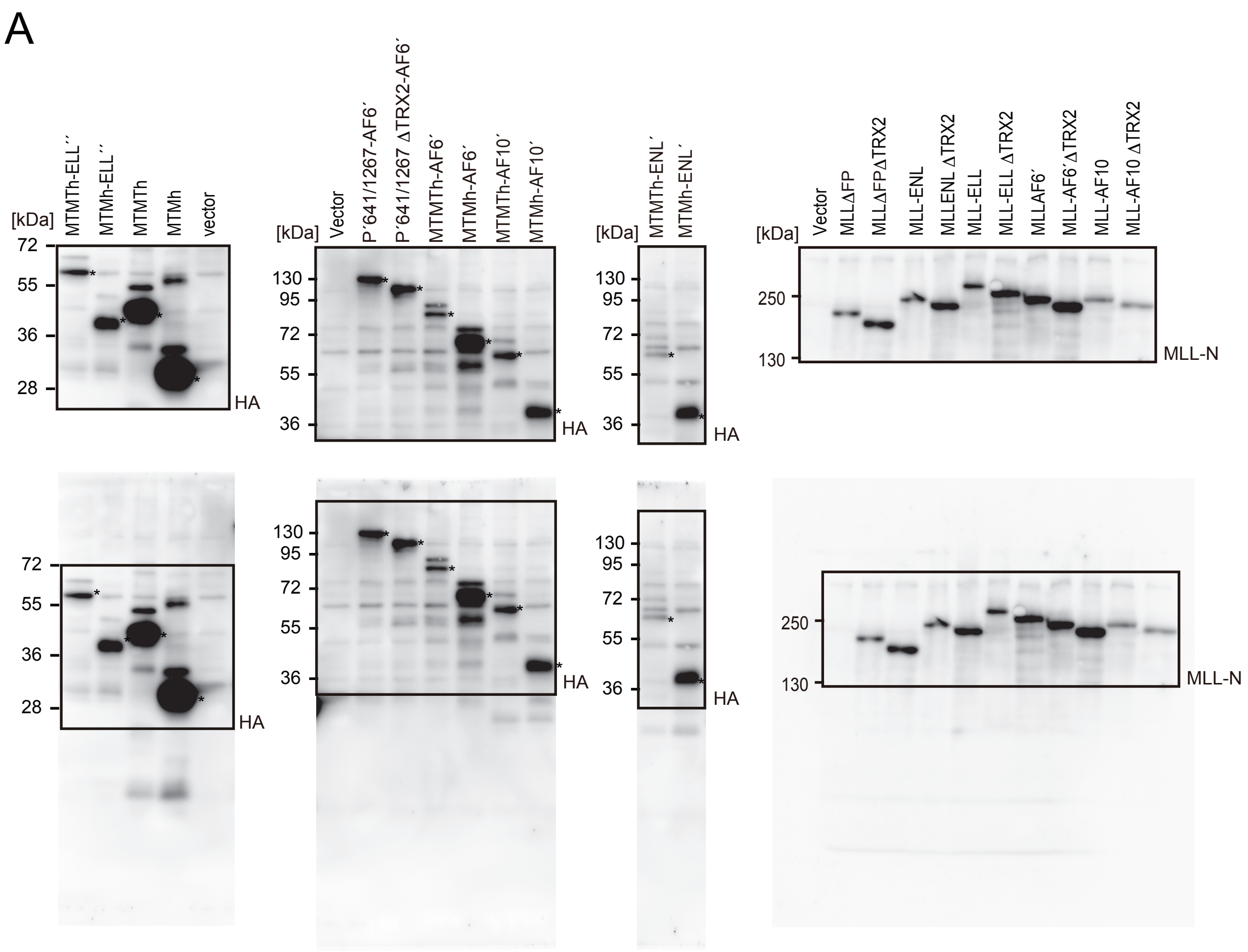

Figure 2-figure supplement 1

A

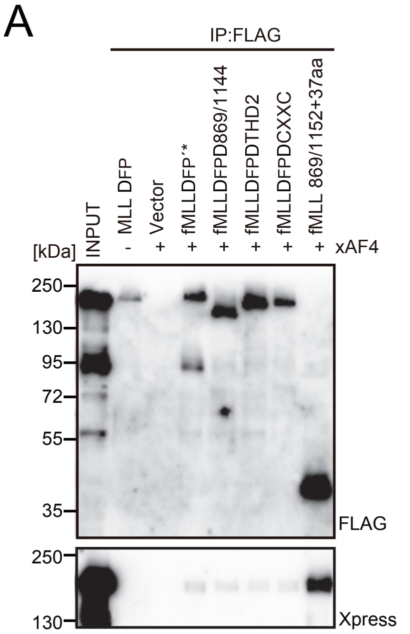

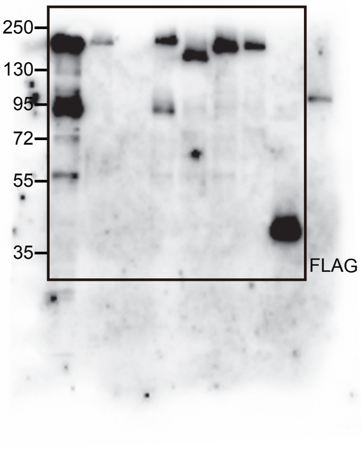

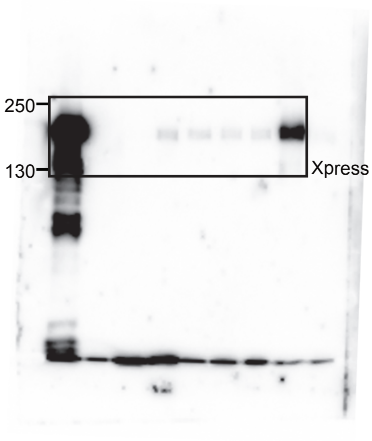

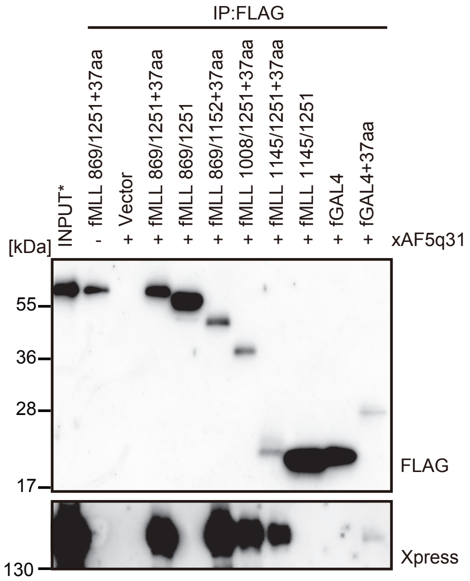

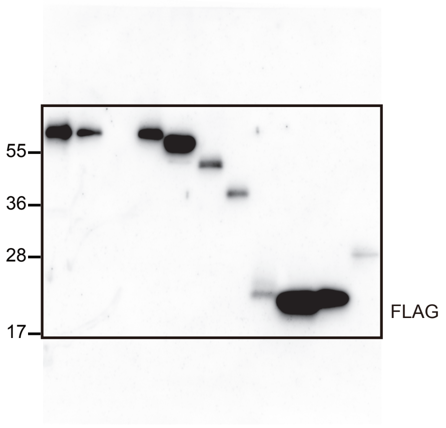

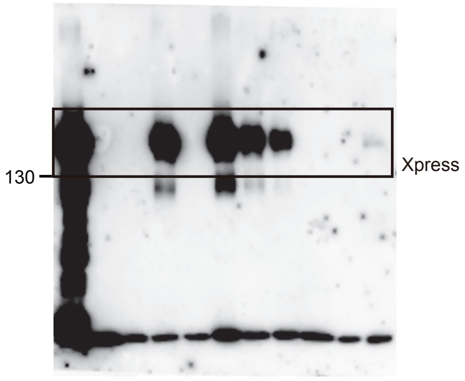

Figure 2-figure supplement 2

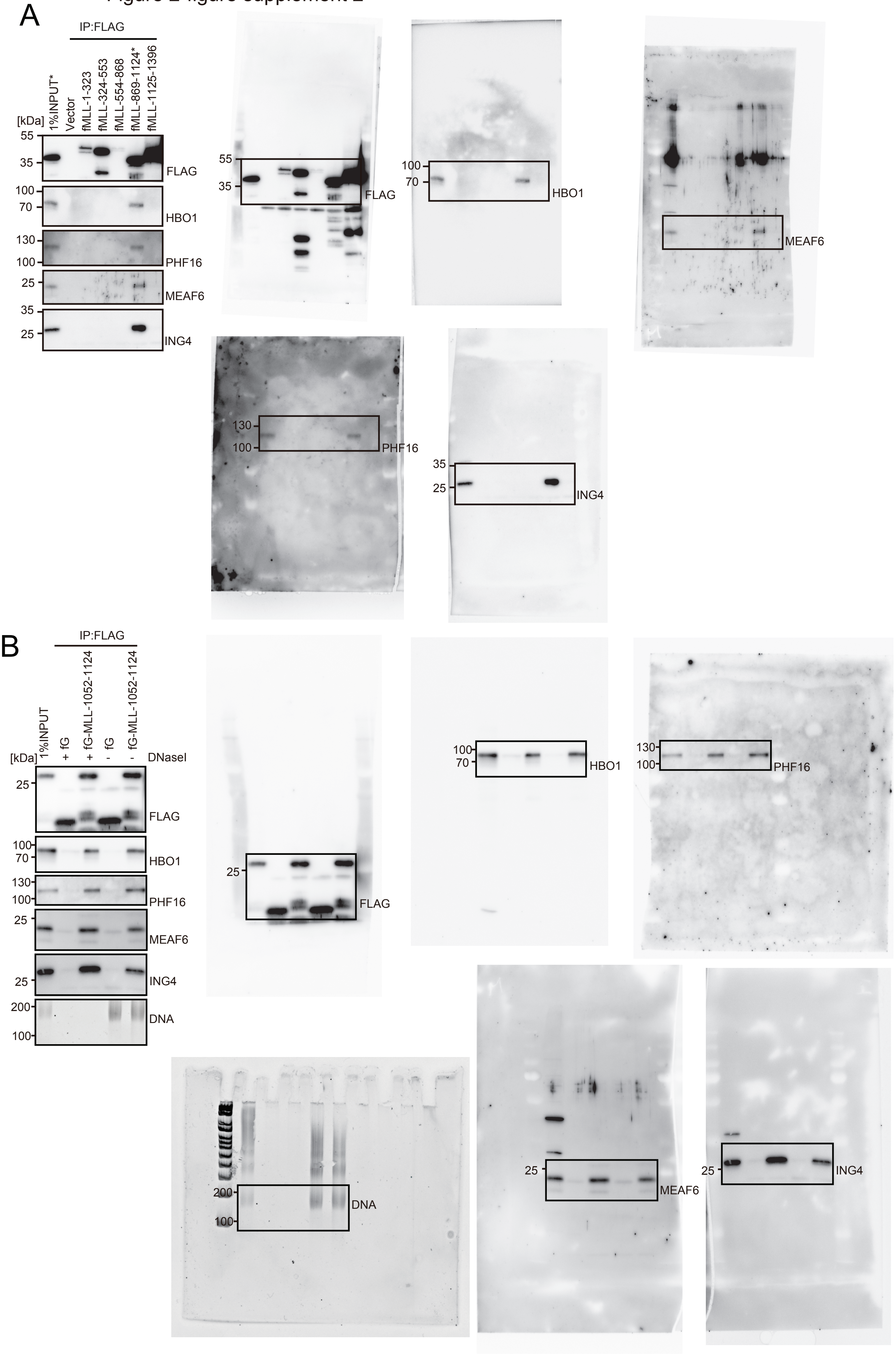

C

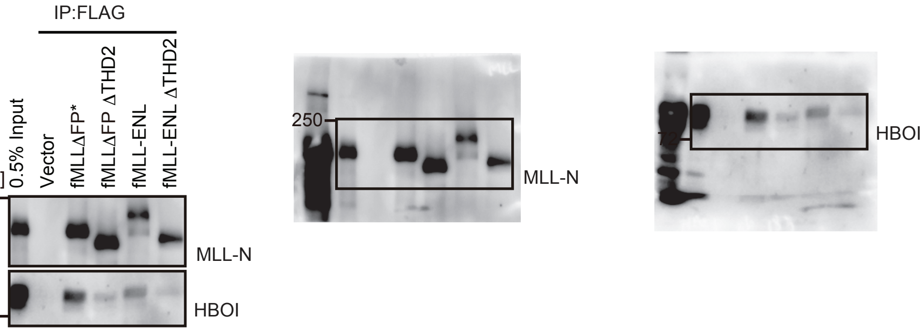

IP:FLAG
0.5% Input
Vector
fMLLΔFP*
fMLLΔFP ΔTHD2
fMLL-ENL
fMLL-ENL ΔTHD2
[kDa]
250
72
MLL-N
HBOI
250
MLL-N
HBOI

D

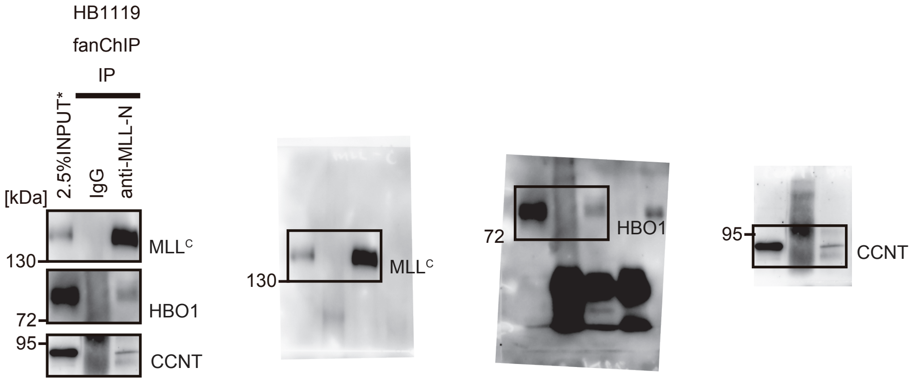

HB1119
fanChIP
IP
2.5%INPUT*
IgG
anti-MLL-N
[kDa]
130
72
95
MLLC
HBO1
CCNT
130
MLLC
72
HBO1
95
CCNT

E

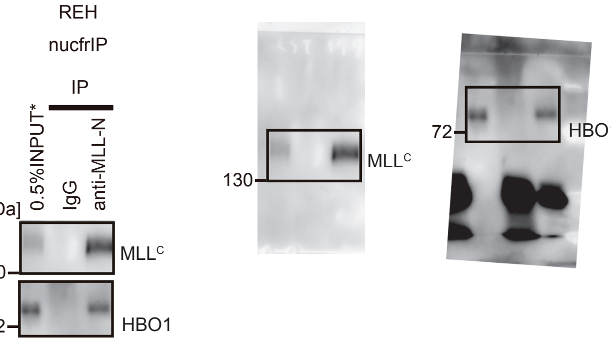

REH
nucfrIP
IP
0.5%INPUT*
IgG
anti-MLL-N
[kDa]
130
72
MLLC
HBO1
130
MLLC
72
HBO1

Figure 4-figure supplement 1

A

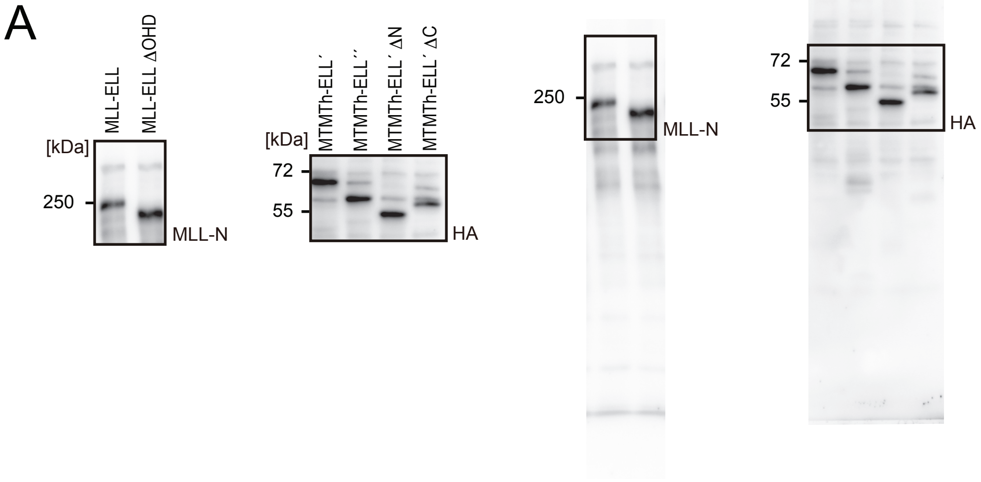

C

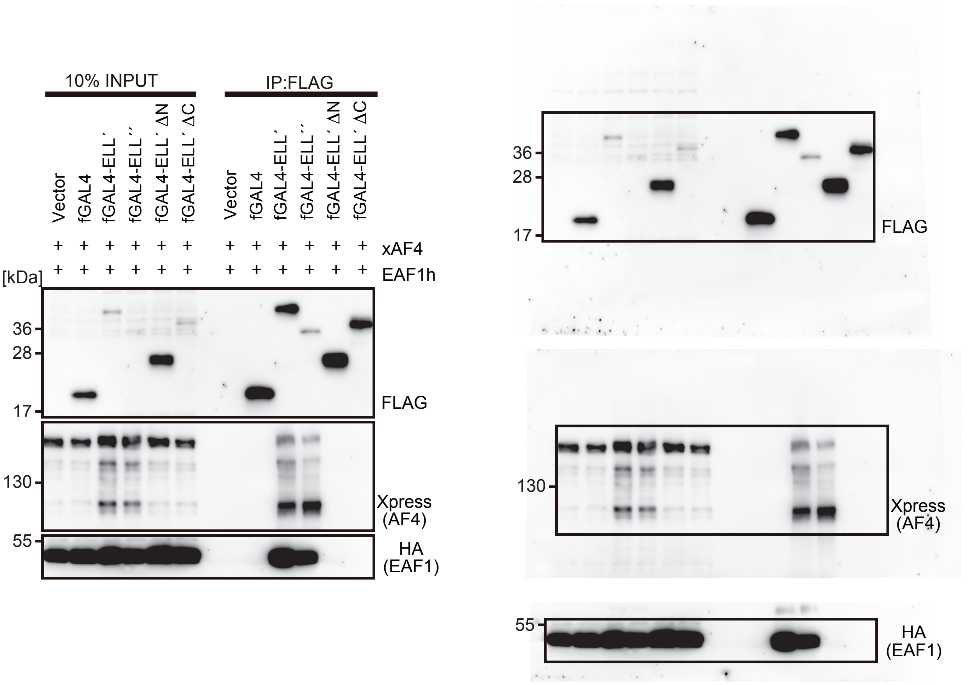

Figure 5-figure supplement 1

A

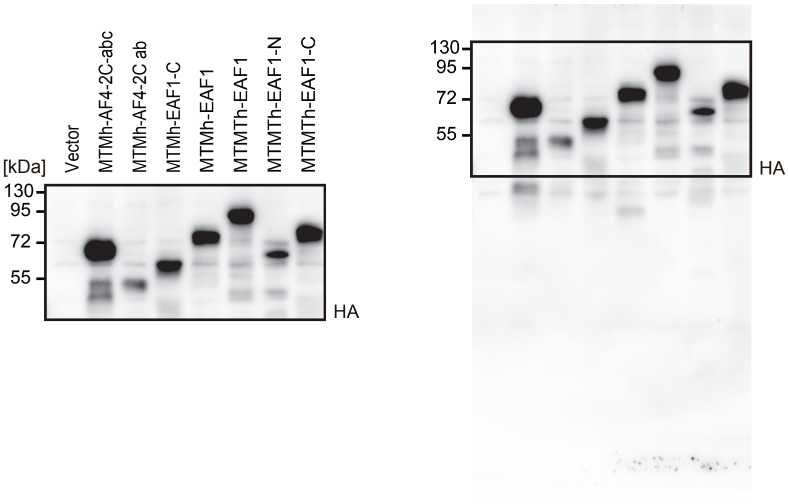

Figure 6-figure supplement 1

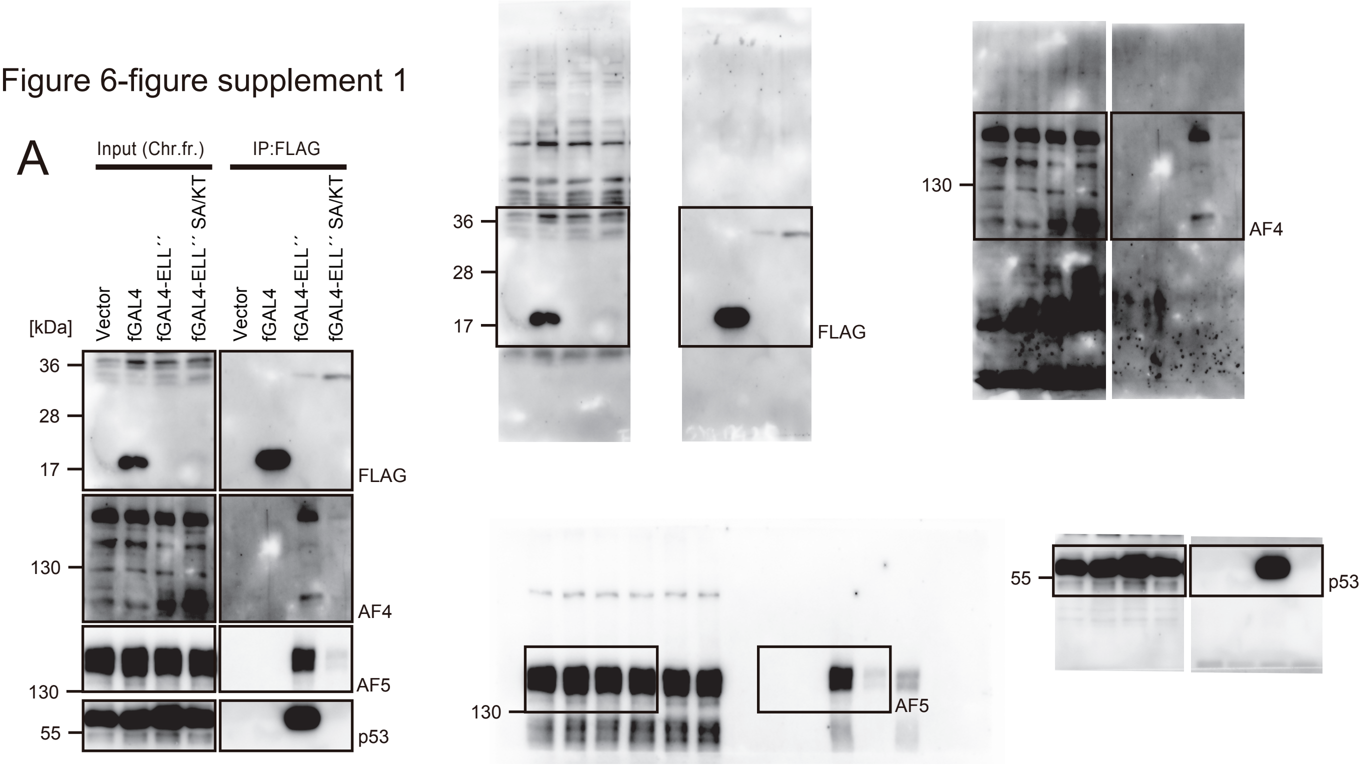

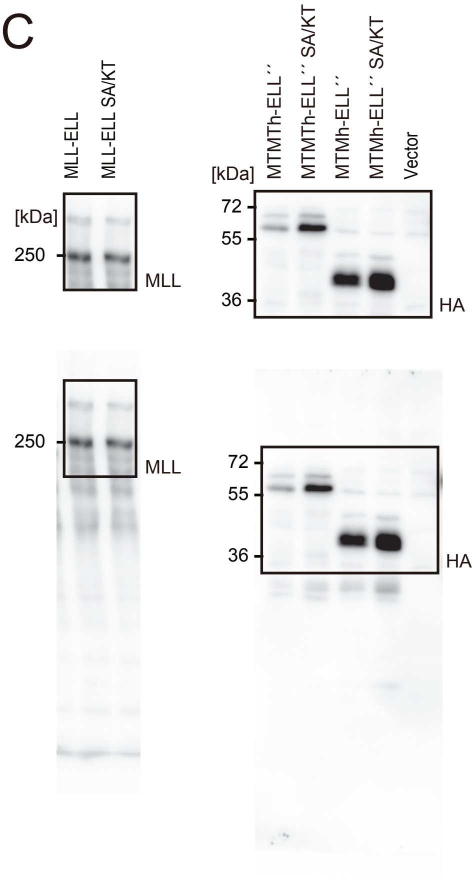

Supplement: Source data 1. [file elife-65872-data1.pdf]
