# Supplementary material for: HBO1-MLL interaction promotes AF4/ENL/P-TEFb-mediated leukemogenesis
Source: eLife. 2021 Aug 25;10:e65872. doi: 10.7554/eLife.65872 (PMC8387021; doi:10.7554/eLife.65872)
Supplement: Supplementary file 1. [file elife-65872-supp1.docx]

Supplementary file 1 : Sequence data

HBO1-MLL interaction promotes AF4/ENL/P-TEFb-mediated leukemogenesis

Satoshi Takahashi, Akinori Kanai, Hiroshi Okuda, Ryo Miyamoto, Yosuke Komata, Takeshi Kawamura, Hirotaka Matsui, Toshiya Inaba, Akifumi Takaori-Kondo, Akihiko Yokoyama

Supplementary file 1a. sgRNA sequences for mouse genes

Supplementary file 1b. sgRNA sequences for human genes

Supplementary file 1c. Custom qPCR probe/primer sequences

Supplementary file 1d. Accession numbers of the NGS data

**Supplementary file 1a. sgRNA sequences for mouse genes**

| sgRNA | Sequence |
| --- | --- |
| Kat7#6 | AGCCGCCGGCAATGCCGCGA |
| Kat7#8 | CCTCTCAGCTGACGAATGCA |
| Eaf1#1 | ATTGTCTTCGCCTGCGCTGC |
| Eaf1#2 | TTCCCTTTGAACACCGTCAT |
| Eaf1#3 | GCTGTCGTCGTCACTTCCCG |
| Trp53#1 | AGTGAAGCCCTCCGAGTGTC |
| Trp53#2 | AACAGATCGTCCATGCAGTG |
| Trp53#3 | TGAGGGCTTACCATCACCAT |
| Kmt2a#1 | GATCGCCGCCCTTTCTTCAC |
| Kmt2a#2 | AGGCGGTGCGGACAATGTCC |
| Rpa3 | GCTGGCGTTGACGCGCGCTT |
| Ren | GGATGATAACTGGTCCGCAG |

**Supplementary file 1b. sgRNA sequences for human genes**

| sgRNA | Sequence |
| --- | --- |
| KAT7#2 | GCCGCTATGAGCTTGATACC |
| KAT7#4 | GATCTGAGAGTGGACGTTCT |
| RPA3#6 | CAGGCTTGTCGATGAATTGAGCTAGCATGC |

**Supplementary file 1c. Custom qPCR probe/primer sequences**

| Target name | Forward | Reverse | Reporter |
| --- | --- | --- | --- |
| EVI-1 TSS | GCTGCGGAGGATCTGAAAGG | CTCCTTCCCAGTTCCAATGGG | CAGGAGGAGGAGAGTTT |
| RPL13A-TSS | GCCGGGTGGGATCCA | GCTCCGCAAAACATGCAAGA | CAGAGAGGGTGCGACCCCATT |
| CDKN2C- TSS | GGCGGCTGCCCTGT | CCCGGTGCCACTTTGC | CTGTGCCCCTTTGCTG |
| MYC-TSS | CCGGCTAGGGTGGAAGAG | GAGGCGAAGCCCCCTATTC | CAGGACGCCCGCAGCG |
| LUC-pr | CGGCGCCATTCTATCCTCTAG | AGGGCGTATCTCTTCATAGCCTTAT | CTCCAGCGGTTCCATC |
| CD4- pre-TSS | TGTCCGAGCAAGGGATGATATTG | CCAAGTCACTCTGCACTACCA | ACTGCCACCATGCCAAT |
| CD4- TSS | GCAGCGGGCAAGAAAGAC | ACGTTCCCCTCCCTCTCA | CCGAGTCTGACCACCTTAC |
| CD4- post-TSS | GGACTCACTGTCCCTCCTGAA | CTTCAAGGCCATGAGGTCTCA | TCAGCCTTTCCGCCCTC |
| CD4 last exon | GGCTGGCAGTGACAGAACT | CCGGCACTTGGTAATCAATCATCTA | CTGGTTCTGGTCTATAAACT |
| HOXA7 pre-TSS | GCCTTCCCCGTCTGGAT | ACTCTGCCCAAGTCTTCTCTCA | CAGGCCGGACTTAGAC |
| HOXA7 TSS | GACGCCTACGGCAACCT | GCCTTTGGCGAGGTCACT | CCCTGCGCCTCCTAC |
| HOXA7 post-TSS | TGCCAGGGTCCATTTCAAGATG | CCCTCATCCCCAGGACCTT | CTCTGTCCTCATTCCC |
| HOXA7-last exon | GCACCACCCTCCCAGAC | CCAGCCAGCACAAAATAGGTAGTTT | CCGCAAGAAAGTGAATCT |

**Supplementary file 1d. Accession numbers of the NGS data**

| **Sample name** | **DRA accession number** | **Sample ID** | **GEA accession number** |
| --- | --- | --- | --- |
| HB1119-fanChIP-INPUT | DRA010818 | SAMD00247188 | E-GEAD-401 |
| HB1119-fanChIP-INPUT#2 | DRA012472 | SAMD00393831 | E-GEAD-445 |
| HB1119-fanChIP-MLLn | DRA004871 | SAMD00055685 | E-GEAD-319 |
| HB1119-fanChIP-MLLn#2 | DRA012472 | SAMD00393832 | E-GEAD-445 |
| HB1119-fanChIP-HBO1 | DRA010818 | SAMD00247189 | E-GEAD-401 |
| HB1119-fanChIP-HBO1#2 | DRA012472 | SAMD00393833 | E-GEAD-445 |
| HB1119-fanChIP-PHF16 | DRA010818 | SAMD00247190 | E-GEAD-401 |
| HB1119-fanChIP-PHF16#2 | DRA012472 | SAMD00393834 | E-GEAD-445 |
| HB1119-fanChIP- MEAF6 | DRA010818 | SAMD00247191 | E-GEAD-401 |
| HB1119-fanChIP- MEAF6#2 | DRA012472 | SAMD00393835 | E-GEAD-445 |
| HB1119-fanChIP- ING4 | DRA010818 | SAMD00247192 | E-GEAD-401 |
| HB1119-fanChIP- ING4#2 | DRA012472 | SAMD00393836 | E-GEAD-445 |
| HB1119-fanChIP-RNAP2 Ser5-P | DRA010818 | SAMD00247193 | E-GEAD-401 |
| HB1119-fanChIP-RNAP2 Ser5-P#2 | DRA012472 | SAMD00393837 | E-GEAD-445 |
| HB1119-fanChIP-RNAP2 non-P | DRA010818 | SAMD00247194 | E-GEAD-401 |
| HB1119-fanChIP-RNAP2 non-P#2 | DRA012472 | SAMD00393838 | E-GEAD-445 |
| 293T-fanChIP-INPUT | DRA004872 | SAMD00055699 | E-GEAD-320 |
| 293T-fanChIP-INPUT#2 | DRA012473 | SAMD00393839 | E-GEAD-446 |
| 293T-fanChIP-H3K4me3 | DRA004872 | SAMD00055702 | E-GEAD-320 |
| 293T-fanChIP-MLL | DRA010819 | SAMD00247195 | E-GEAD-402 |
| 293T-fanChIP-MLL#2 | DRA012473 | SAMD00393840 | E-GEAD-446 |
| 293T-fanChIP-HBO1 | DRA010819 | SAMD00247196 | E-GEAD-402 |
| 293T-fanChIP-HBO1#2 | DRA012473 | SAMD00393841 | E-GEAD-446 |
| 293T-fanChIP-PHF16 | DRA010819 | SAMD00247197 | E-GEAD-402 |
| 293T-fanChIP-PHF16#2 | DRA012473 | SAMD00393842 | E-GEAD-446 |
| 293T-fanChIP-ING4 | DRA010819 | SAMD00247198 | E-GEAD-402 |
| 293T-fanChIP-ING4#2 | DRA012473 | SAMD00393843 | E-GEAD-446 |
| 293T-fanChIP-MEAF6 | DRA010819 | SAMD00247199 | E-GEAD-402 |
| 293T-fanChIP-MEAF6#2 | DRA012473 | SAMD00393844 | E-GEAD-446 |
| 293T-fanChIP-MOZ | DRA008732 | SAMD00180127 | E-GEAD-322 |
| 293T-fanChIP-ENL | DRA010819 | SAMD00247200 | E-GEAD-402 |
| 293T-fanChIP-ENL#2 | DRA012473 | SAMD00393845 | E-GEAD-446 |
| 293T-fanChIP-H3K18ac | DRA008732 | SAMD00180131 | E-GEAD-322 |
| 293T-fanChIP-DOT1L | DRA004872 | SAMD00055697 | E-GEAD-320 |
| 293T-fanChIP-AF17 | DRA004872 | SAMD00055710 | E-GEAD-320 |
| 293T-fanChIP-AF4 | DRA004872 | SAMD00055708 | E-GEAD-320 |
| 293T-fanChIP-CCNT1 | DRA008732 | SAMD00180125 | E-GEAD-322 |
| 293T-fanChIP-TAF1C | DRA004872 | SAMD00055705 | E-GEAD-320 |
| 293T-fanChIP-RNAP2 non-P | DRA010819 | SAMD00247201 | E-GEAD-402 |
| 293T-fanChIP-RNAP2 non-P#2 | DRA012473 | SAMD00393846 | E-GEAD-446 |
| 293T-fanChIP-RNAP2 Ser5-P | DRA004872 | SAMD00055704 | E-GEAD-320 |
| 293T-fanChIP-RNAP2 Ser5-P#2 | DRA012473 | SAMD00393847 | E-GEAD-446 |
| 293T-CIRA-INPUT | DRA008734 | SAMD00180208 | E-GEAD-324 |
| 293T-CIRA-unMe CpGs | DRA008734 | SAMD00180209 | E-GEAD-324 |
| 293T-RNA | DRA004874 | SAMD00055715 | E-GEAD-321 |
