# Supplementary material for: HBO1-MLL interaction promotes AF4/ENL/P-TEFb-mediated leukemogenesis
Source: eLife. 2021 Aug 25;10:e65872. doi: 10.7554/eLife.65872 (PMC8387021; doi:10.7554/eLife.65872)
Supplement: Transparent reporting form [file elife-65872-transrepform.docx]

***eLife’s* transparent reporting form**

We encourage authors to provide detailed information *within their submission* to facilitate the interpretation and replication of experiments. Authors can upload supporting documentation to indicate the use of appropriate reporting guidelines for health-related research (see [EQUATOR Network](http://www.equator-network.org/%20)), life science research (see the [BioSharing Information Resource](https://biosharing.org/)), or the [ARRIVE guidelines](http://www.plosbiology.org/article/info:doi/10.1371/journal.pbio.1000412) for reporting work involving animal research. Where applicable, authors should refer to any relevant reporting standards documents in this form.

If you have any questions, please consult our Journal Policies and/or contact us: [editorial@elifesciences.org](mailto:editorial@elifesciences.org).

**Sample-size estimation**

- You should state whether an appropriate sample size was computed when the study was being designed
- You should state the statistical method of sample size computation and any required assumptions
- If no explicit power analysis was used, you should describe how you decided what sample (replicate) size (number) to use

Please outline where this information can be found within the submission (e.g., sections or figure legends), or explain why this information doesn’t apply to your submission:

The chosen sample sizes are basically based on the numbers used for previous publications. In transplantation assay, donor cells underwent gene transduction/modification were injected into 5 mice/group. This procedure was duplicated, usually giving final sample size of N = 10/group. In some cases, larger sample size (N=20/group) was used to precisely determine the penetrance of certain types of leukemia (Figure 6D)

**Replicates**

- You should report how often each experiment was performed
- You should include a definition of biological versus technical replication
- The data obtained should be provided and sufficient information should be provided to indicate the number of independent biological and/or technical replicates
- If you encountered any outliers, you should describe how these were handled
- Criteria for exclusion/inclusion of data should be clearly stated
- High-throughput sequence data should be uploaded before submission, with a private link for reviewers provided (these are available from both GEO and ArrayExpress)

Please outline where this information can be found within the submission (e.g., sections or figure legends), or explain why this information doesn’t apply to your submission:

Numbers of experiments and whether they are biological or technical replicates are indicated in the figure legends. All of the IP -western, ChIP-qPCR, RT-qPCR experiments were performed at least twice and confirmed their reproducibility. Myeloid progenitor transformation assay was performed at least three times (≥3 biological replicates). ChIP-seq analysis was mostly performed twice as in described in Supplemental table 4 in the Supplemental file 1.

All of the NGS data have been deposited to the DDBJ archive in the following folder.

ftp://ftp.ddbj.nig.ac.jp/ddbj_database/dra/fastq/

ftp://ftp.ddbj.nig.ac.jp/ddbj_database/gea/experiment/E-GEAD-000/

The BigWig files of E-GEAD-401, 402, 445, and 446) are in the following GEA reviewer access system.

https://ddbj.nig.ac.jp/gea/reviewer/login

Access key for E-GEAD-401: 6dp0gJHk2Wqb2ziRaj66

Access key for E-GEAD-402: jpQaPvkU2agSoHCr9czU

Access key for E-GEAD-445: FvZYhju3GxWK0QnkiVNG

Access key for E-GEAD-446: v88kfT9gEL9ByChUdRVD

The mass spectrometry data were deposited to JPOST repository (ID:JPST001262; PXID:PXD027400).

For preview of the mass spectrometry data, the data can be obtained via below URL with the access key.

<https://repository.jpostdb.org/preview/129878960660f62c93e22ae>

Access key:6025

**Statistical reporting**

- Statistical analysis methods should be described and justified
- Raw data should be presented in figures whenever informative to do so (typically when N per group is less than 10)
- For each experiment, you should identify the statistical tests used, exact values of N, definitions of center, methods of multiple test correction, and dispersion and precision measures (e.g., mean, median, SD, SEM, confidence intervals; and, for the major substantive results, a measure of effect size (e.g., Pearson's r, Cohen's d)
- Report exact p-values wherever possible alongside the summary statistics and 95% confidence intervals. These should be reported for all key questions and not only when the p-value is less than 0.05.

Please outline where this information can be found within the submission (e.g., sections or figure legends), or explain why this information doesn’t apply to your submission:

Statistical methods used are indicated in the figure legends. Data are represented as mean ± SD with the number of replicates. For multiple comparisons for in vitro experiments, ANOVA followed by Dunnett’s test was used. For correlation analysis on the ChIP-seq data, Spearman’s rank correlation coefficient is shown. For in vivo experiments, log-rank test followed by Bonferroni correction was used.

(For large datasets, or papers with a very large number of statistical tests, you may upload a single table file with tests, Ns, etc., with reference to sections in the manuscript.)

**Group allocation**

- Indicate how samples were allocated into experimental groups (in the case of clinical studies, please specify allocation to treatment method); if randomization was used, please also state if restricted randomization was applied
- Indicate if masking was used during group allocation, data collection and/or data analysis

Please outline where this information can be found within the submission (e.g., sections or figure legends), or explain why this information doesn’t apply to your submission:

In transplantation assay, irradiated recipient mice were allocated into experimental groups at random. After injection, mice were kept in a cage of the same experimental group. If needed to increase the sample size of certain groups, more recipient mice were newly allocated only for the groups. No blinding was used.

**Additional data files (“source data”)**

- We encourage you to upload relevant additional data files, such as numerical data that are represented as a graph in a figure, or as a summary table
- Where provided, these should be in the most useful format, and they can be uploaded as “Source data” files linked to a main figure or table
- Include model definition files including the full list of parameters used
- Include code used for data analysis (e.g., R, MatLab)
- Avoid stating that data files are “available upon request”

Please indicate the figures or tables for which source data files have been provided:
